# The Mediating Effect of Professional Benefits on Nurse's Work Stressor and Humanistic Practice Ability of ICU Nurses

**DOI:** 10.1002/nop2.70449

**Published:** 2026-06-11

**Authors:** Yajuan Zhang, Kaihong Fei, Wenqi Tang, Zixin Wang, Chenliang Pan, Zhipeng Wang, Lili Ma, Qunfeng Lu

**Affiliations:** ^1^ Department of Nursing Shanghai Sixth People's Hospital Affiliated to Shanghai Jiao Tong University School of Medicine Shanghai China; ^2^ Department of Nursing Shanghai General Hospital, Shanghai Jiao Tong University, School of Medicine Shanghai China; ^3^ Department of Nursing Shanghai East Hospital, School of Medicine, Tongji University Shanghai China

**Keywords:** humanistic practice ability, ICU, mediating effect, nurse, professional benefit, work stressor

## Abstract

**Aim:**

To Explore the Function and Mechanism of Professional Benefits on ICU Nurses' Stressors and Their Humanistic Practice Ability.

**Design:**

A cross‐sectional study was conducted.

**Methods:**

A total of 257 ICU nurses from nine tertiary and five secondary hospitals in Shanghai were recruited from December 2023 to January 2024 using convenience sampling. The study utilised a general data questionnaire, a humanistic practice ability scale, a nurse's work stressor scale, and a professional benefit scale for investigation and analysis.

**Results:**

The scores of ICU nurses' professional benefits, nurses' work stressors, and humanistic practice ability were 61.33 ± 22.21, 70.13 ± 15.70, and 105.96 ± 18.26. Nurse's work stressors were negatively correlated with humanistic practice ability and professional benefits; professional benefits were positively correlated with humanistic practice ability. Professional benefit played a mediating role between nurses' work stressors and humanistic practice ability.

**Conclusions:**

ICU nurses' professional benefits were the mediating variable between stress and humanistic practice ability. ICU nursing managers should pay attention to optimising the working environment of ICU nurses to reduce work pressure, improve nurses' professional benefits, promote ICU nurses' humanistic quality and humanistic practice ability, and better meet the needs of severe patients.

**Implications for the Profession:**

We suggest that medical institutions and managers should implement targeted strategies to enhance nurses' perception of professional benefits and working environment to enhance their perception of career benefits. Therefore, it is essential to identify the current situation and relationship between ICU nurses' professional benefits, nurse's work stressors and humanistic practice ability.

**Patient or Public Contribution:**

No patient or public contribution.

## 1 Introduction

In recent years, critical care has assumed an increasingly vital role in the treatment of patients experiencing severe trauma, major surgery, multi‐organ failure, and in the prevention and management of disaster events and public health crises. Consequently, there are heightened demands for the emergency skills, knowledge base, stress resilience, and both physical and mental qualities of ICU nurses (Ross et al. 2023). The National Nursing Development Plan of China (2021–2025) further emphasizes the need to enhance the motivation of critical care nurses, strengthen the humanistic aspects of nursing care, and optimize nursing services (National Health Commission 2022).

Humanistic practice ability refers to nurses' integrated application of humanistic knowledge, communication techniques, and ethical reasoning in clinical work (National Health Commission 2022). This capability not only requires nurses to possess solid medical knowledge but also demands excellent interpersonal skills and a high sense of professional responsibility. In the ICU, humanistic practice skills are particularly crucial, as ICU nurses experience significant pressure that not only demands higher levels of critical professional competence but also underscores the importance of these skills for better serving severely ill patients (Gai et al. 2023). The humanistic practice ability of ICU nurses refers to their capacity to apply humanistic knowledge and skills when addressing challenges in severe clinical care. This ability is crucial for improving nursing quality, fostering harmonious relationships between nurses and patients, reducing medical disputes, and advancing the medical industry (Zhang et al. 2021). However, there is a shortage of qualified ICU nurses, which directly impacts the delivery and quality of critical care services. In China, the number of ICU nurses remains insufficient to meet the growing demand for intensive care, especially in tertiary hospitals where the complexity and severity of cases are high (Zhang et al. 2024). The ICU nursing role is characterised by high skill requirements, heavy workloads, and elevated psychological stress, contributing to job dissatisfaction and a decline in both retention rates and the quality of humanistic care (Nasirizad Moghadam et al. 2021).

Moreover, studies conducted in Xiamen and other regions of China have shown that ICU nurse work stress is associated with reduced patient satisfaction, increased burnout, and a higher incidence of nursing errors, suggesting a negative impact on the healthcare delivery system (Liang and Fan 2017). These outcomes underscore the urgency of exploring mechanisms to mitigate stress and enhance care quality in the ICU.

While prior research has investigated the relationship between work stress and job satisfaction, or between professional benefit and practice ability (Parizad et al. 2022), there remains a significant gap in comprehensively understanding the triadic relationship among work stressors, professional benefit, and humanistic practice ability—particularly in the ICU context. Notably, few studies have examined this mediating effect in this pathway, especially within China's healthcare system.

The Job Demands–Resources (JDR) model (Demerouti et al. 2001) can illustrate the potential mediating role of professional benefits in the relationship between work stressors and humanistic practice ability among intensive care unit (ICU) nurses. ICU nurses are routinely exposed to high‐intensity work environments characterised by heavy workloads, time pressure, emotional labor, ethical dilemmas, shift work, and frequent encounters with critically ill or dying patients. Such work stressors represent substantial job demands that consume nurses' emotional and cognitive resources, thereby increasing the risk of adverse occupational outcomes and diminishing the quality of care delivery.

Work stressors are hypothesized to directly and negatively affect humanistic practice ability. Humanistic practice ability refers to nurses' capacity to deliver compassionate, patient‐centered, and ethically grounded care, including empathy, respect for patient dignity, effective communication, and emotional support. Prolonged exposure to excessive stress may lead to emotional exhaustion and depersonalization, causing nurses to prioritise task completion over relational engagement and thereby weakening humanistic care behaviours.

Professional benefit is proposed as a key mediator that explains the mechanism linking work stressors to humanistic practice ability. Professional benefit reflects nurses' perceived positive gains from their professional role, such as a sense of professional identity, personal growth, meaningful work, and social recognition. Grounded in the Conservation of Resources theory and positive psychology, professional benefit is conceptualised as a psychological resource that enhances resilience and intrinsic motivation (Hobfoll 1989). High levels of work stressors may undermine nurses' perceptions of professional benefit by reducing feelings of accomplishment and professional value (Prapanjaroensin et al. 2017).

In contrast, nurses with higher perceived professional benefit are more likely to maintain empathic engagement and compassionate practice despite stressful working conditions (Albrecht 1982). A strong sense of professional benefit may enable nurses to reinterpret occupational stress as meaningful and worthwhile, thereby preserving emotional availability and reinforcing humanistic values in clinical care (Albrecht 1982). Accordingly, professional benefit is expected to be positively associated with humanistic practice.

Given the nature and theory of work stress, professional benefit, and humanistic practice ability—all of which can be measured using validated standardised questionnaires—this study is positioned to fill a critical knowledge gap. It not only explores the status and interrelationships of these variables but also applies structural equation modelling (SEM) to examine the mediating role of professional benefits, thereby providing a theoretical and empirical basis for improving ICU nursing management.

This study aimed to explore the function and mechanism of professional benefits on ICU nurses' stressors and their humanistic practice ability by examining the following research questions: (1) What is the current status of professional benefits, stress levels, and humanistic practice ability among ICU nurses in China? (2) What is the mediating role of humanistic practice ability in the relationship between professional benefits and stress among ICU nurses?

## 2 Methods

### 2.1 Design, Sample Size, and Recruitment

This study is a multi‐centered cross‐sectional survey research. From December 2023 to January 2024, the research group used the convenient sampling method to investigate 257 ICU nurses from nine tertiary hospitals and five secondary hospitals in Shanghai. Inclusion criteria: (1) registered nurse; (2) currently working in the ICU and working in the ICU at least 1 year; (3) signed informed consent and voluntarily participated in this study. Exclusion criteria: nurses who cannot complete the questionnaire due to sick leave, personal leave, etc.; (4) training nurse. In this study, according to the requirements of Structural Equation Model (Wu 2010), the recommended sample size for structural model construction is at least 200, with an additional 5–10 participants required for each extra variable. Three variables were included in the model; by calculating the sample size, it should be at least 215–230 samples. The research group first communicated with the nursing department and ICU to explain the aims and process of the study. After obtaining their consent, the head nurse sent a private letter to explain the purpose and significance of the study to all the nurses who met the inclusion criteria. After obtaining informed consent from every nurse, the questionnaire was sent to each participant. In this study, a total of 270 questionnaires were distributed, and 257 valid questionnaires were collected, with an effective rate of 95.18%.

### 2.2 Instruments With Validity and Reliability

#### 2.2.1 General Data Questionnaire

On the basis of full access to literature review and group discussion, 14 variables including gender, age, marital status, ethnic, whether the only child, procreation status, educational background, ICU working years, professional title, administrative position, monthly income, hospital level, numbers of night shift and whether to accept humanities related training course were included in this study.

#### 2.2.2 Professional Benefit Scale

The professional benefit scale (Hu 2013) used in this study included 5 dimensions and 33 items including Positive career perception, family and friend recognition, team belonging, good nurse–patient relationships, and self‐growth, and the Likert 5‐point scoring method was adopted, with responses ranging from strongly disagree to strongly agree and assigned 1–5 points; the higher score indicates a stronger sense of professional benefits. The scale had good reliability and validity, and the total Cronbach *α* coefficient was 0.958 and Cronbach *α* coefficients of each dimension were 0.821–0.893 (Hu 2013). In this study, the total Cronbach *α* coefficient of the scale was 0.983, and the Cronbach α coefficient of each dimension was 0.901–0.953.

#### 2.2.3 Nurse's Work Stressor Scale

The nurse's work stressors scale, developed by Li and Liu (Li and Liu 2000), was used in this study. The scale had 5 dimensions and 35 items and was scored on a 4‐point Likert scale, from never to almost every day, with points assigned in ascending order from 1 to 4. NJSS included 5 dimensions: nursing profession and work, workload and distribution, working environment and resources, patient care and management, and interpersonal relationship respectively. The higher the NJSS score, the greater the degree of work stress of nurses. Higher scores indicate greater pressure. The scale had good reliability and validity. The total Cronbach *α* coefficient was 0.94, and the Cronbach *α* coefficient for each dimension ranged from 0.80 to 0.89 (Li and Liu 2000). In this study, the total Cronbach *α* coefficient was 0.955, and the Cronbach *α* coefficient for each dimension was 0.717–0.902.

#### 2.2.4 Humanistic Practice Ability Scale

The Humanistic Practice Ability Scale was developed by Yan (Yan 2016), which includes 5 dimensions and 26 items: (a) humanistic care practical ability (10 items), (b) nursing communication ability (6 items), (c) ethics and legal application ability (3 items), (d) self‐management ability (3 items), and (e) psychological adjustment ability (4 items). The total Cronbach α coefficient of the scale was 0.913, and the Cronbach α coefficient of each dimension was 0.715–0.877. The content validity of the total scale was 0.908, and the content validity of each dimension was 0.84–1.00 (Yan 2016). The scale adopted the Likert 5 scoring method and assigns the value of 1–5 points from very inconsistent ~ very consistent. The higher the score, the better the humanistic practice ability. In this study, the total Cronbach *α* coefficient of the scale was 0.986, and the Cronbach *α* coefficient of each dimension was 0.861–0.981.

### 2.3 Data Collection and Analysis

Data were collected through a structured questionnaire survey administered to ICU nurses between December 2023 and January 2024. Participants were recruited through convenience sampling in nine tertiary and five secondary hospitals in Shanghai. After obtaining informed consent, participants completed four instruments: the general demographic questionnaire, the Professional Benefit Scale, the Nurse's Work Stressor Scale, and the Humanistic Practice Ability Scale. All questionnaires were self‐administered and returned anonymously to ensure confidentiality and data integrity.

Data analysis was performed using SPSS 22.0 and AMOS 24.0. All measurement data conforming to a normal distribution are expressed as *x̄* ± *s*, while count data is described using frequencies and percentages. If the bivariate variables related to professional benefit, work stressor, and humanistic practice ability meet the criteria for normal distribution, Pearson's correlation will be employed for analysis. Conversely, if these criteria are not met, Spearman's correlation will be utilised. Additionally, AMOS 24.0 is used to construct a structural equation model, and the Bootstrap method using 5000 bootstrap samples and 95% confidence intervals is applied to assess and confirm the mediating role of professional benefits in the relationships between humanistic care experience and work stressors. It is important to note that, as a cross‐sectional study, the identified mediation model illustrates potential associations and pathways based on theoretical reasoning but does not establish causality. A *p*‐value of less than 0.05 will denote statistical significance of the observed differences.

### 2.4 Ethical Considerations

This study has been approved by the Ethics Committee of the Sixth People's Hospital Affiliated to Shanghai Jiao Tong University School of Medicine (No. 2023‐YS‐135). All methods used in this study were in accordance with the declaration of Helsinki. Purpose and significance of the study were explained to each participated and informed consents were obtained before distributing the questionnaire. Every participant participated in the study voluntarily. We protected their privacy as they filled the questionnaires anonymously and they could choose not to answer questions as their will.

## 3 Results

### 3.1 Participants' Characteristics

A total of 257 ICU nurses were included in the study, consisting of 47 men and 210 women. Among them, 254 were of Han ethnicity and 3 were from ethnic minority groups. Regarding hospital affiliation, 225 nurses worked in tertiary general hospitals, 21 in secondary general hospitals, and 11 in tertiary specialised hospitals. Additional demographic characteristics collected included age, marital status, educational background, ICU working years, professional title, administrative position, monthly income, number of night shifts per month, and whether the participant had received training related to humanistic care. Detailed information is presented in Table 1.

**TABLE 1:** Distribution of general data of the 265 ICU nurses.

Variable	Classification	Number	Proportion
Gender	Male	47	18.3
	Female	210	81.7
Age	≤ 24	27	10.5
	25–30	93	36.2
	31–40	103	40.1
	41–50	28	10.9
	51–60	6	2.3
Nationality	Han nationality	254	98.8
	Other nationality	3	1.2
Marital status	Married	155	60.3
	Unmarried	102	39.7
Only child of the family	Yes	133	51.8
	No	124	48.2
Procreation status	No child	126	49
	One child	105	40.9
	Two children	25	9.7
	Three children or more	1	0.4
Educational background	Junior college education	46	17.9
	Bachelor degree	198	77
	Master degree or higher	13	5.1
ICU working years	< 5 years	88	34.2
	5–9 years	61	23.8
	10–14 years	62	24.1
	15–19 years	26	10.1
	≥ 20 years	20	7.8
Professional title	Elementary level	184	71.6
	Intermediate level	73	28.4
Administrative position	No	216	84
	Internship tutor	21	8.2
	Deputy head nurse	9	3.5
	Head nurse	10	3.9
	Department head nurse or higher	1	0.4
Monthly income	< 5000	8	3.1
	5000–10,000	111	43.2
	10,001–15,000	115	44.7
	> 15,001	23	9.0
Hospital level	Tertiary general hospital	225	87.5
	Secondary general hospital	21	8.2
	Tertiary specialised hospital	11	4.3
Number of night shifts	Less than 5 per month	65	25.3
	5–10 per month	135	52.5
	More than 10 per month	57	22.2
Received humanistic practice‐related training	No	129	50.2
	Yes	128	49.8

### 3.2 The Scores of 257 ICU Nurses Professional Benefits, Work Stressors and Humanistic Practice Ability Were Shown in Table 2

**TABLE 2:** Total scores and scores of each dimension of work stressors, humanistic practice ability and professional benefits of the 257 ICU nurses ($\bar{x}$ ± *s*).

Scale and dimension	Total scores	Average scores
Work stressors	70.13 ± 15.70	2.00 ± 0.45
Nursing specialty	13.56 ± 3.19	1.94 ± 0.46
Workload and time allocation	10.49 ± 2.84	2.10 ± 0.57
Working environment and resources	5.71 ± 1.49	1.90 ± 0.50
Patient care	20.74 ± 5.56	1.89 ± 0.51
Management and interpersonal relationships	19.63 ± 4.62	2.18 ± 0.51
Humanistic practice ability	105.96 ± 18.26	4.08 ± 0.70
Practical ability of humanistic care	40.29 ± 7.66	4.01 ± 0.75
Psychological adjustment ability	16.89 ± 2.84	4.22 ± 0.71
Interpersonal communication skills	24.82 ± 4.34	4.14 ± 0.72
Self‐management ability	12.02 ± 2.24	3.98 ± 0.75
Ethics and legal practice ability	11.93 ± 2.25	4.03 ± 0.77
Professional benefits	61.33 ± 22.21	1.80 ± 0.65
Positive career perception	13.20 ± 4.73	1.89 ± 0.68
Positive nurse–patient relationships	10.75 ± 4.15	1.79 ± 0.69
Familial and peer recognition	10.79 ± 4.14	1.80 ± 0.69
Sense of group belonging	10.33 ± 4.05	1.72 ± 0.67
Self‐growth	16.26 ± 6.36	1.81 ± 0.71

The total score of ICU nurses' work stressors was (70.13 ± 15.70), showing that the nurses faced high work pressure, and the standard deviation of 15.70 indicated a relatively big difference among different nurses. The score of ICU nurses' humanistic practice ability was (105.96 ± 18.26) at a moderate level and the standard deviation was 18.26, indicating a large difference among different individuals; the ICU professional benefit score was (61.33 ± 22.21), which was at a low level, revealing that the feelings between different nursing staff varied greatly.

### 3.3 Correlation Analysis of Professional Benefits, Work Stressors and Humanistic Practice Ability of ICU Nurses in Table 3

**TABLE 3:** Correlation analysis of work stressors, humanistic practice ability and professional benefit scores of 257 ICU nurses (*r* value).

Variables	1	2	3	4	5	6	7	8	9	10	11	12	13	14	15	16	17	18
1. Nursing specialty	1																	
2. Workload and time allocation	0.826*	1																
3. Working environment and resources	0.728*	0.732*	1															
4. Patient care	0.826*	0.812*	0.746*	1														
5. Management and interpersonal relationships	0.628*	0.650*	0.602*	0.671*	1													
6. Work stressors	0.899*	0.897*	0.817*	0.937*	0.834*	1												
7. Practical ability of humanistic care	−0.458*	−0.337*	−0.370*	−0.356*	−0.265*	−0.393*	1											
8. Psychological adjustment ability	−0.379*	−0.283*	−0.323*	−0.286*	−0.229*	−0.327*	0.931*	1										
9. Interpersonal communication skills	−0.397*	−0.319*	−0.372*	−0.341*	−0.268*	−0.373*	0.896*	0.902*	1									
10. Self‐management ability	−0.391*	−0.354*	−0.341*	−0.356*	−0.256*	−0.377*	0.788*	0.776*	0.864*	1								
11. Ethics and legal practice ability	−0.446*	−0.392*	−0.368*	−0.411*	−0.308*	−0.432*	0.779*	0.764*	0.852*	0.879*	1							
12. Humanistic practice ability	−0.448*	−0.353*	−0.381*	−0.369*	−0.280*	−0.404*	0.969*	0.949*	0.964*	0.887*	0.879*	1						
13. Positive career perception	−0.316*	−0.275*	−0.278*	−0.265*	−0.190*	−0.290*	0.409*	0.419*	0.429*	0.428*	0.502*	0.452*	1					
14. Positive nurse–patient relationships	−0.322*	−0.293*	−0.257*	−0.278*	−0.203*	−0.301*	0.402*	0.420*	0.433*	0.442*	0.517*	0.454*	0.852*	1				
15. Familial and peer recognition	−0.330*	−0.310*	−0.270*	−0.288*	−0.202*	−0.310*	0.413*	0.425*	0.443*	0.447*	0.521*	0.463*	0.833*	0.890*	1			
16. Sense of group belonging	−0.309*	−0.255*	−0.250*	−0.247*	−0.147*	−0.263*	0.405*	0.410*	0.419*	0.432*	0.486*	0.446*	0.841*	0.856*	0.877*	1		
17. Self growth	−0.314*	−0.265*	−0.239*	−0.252*	−0.160*	−0.271*	0.397*	0.419*	0.424*	0.432*	0.475*	0.444*	0.838*	0.863*	0.885*	0.907*	1	
18. Professional benefits	−0.335*	−0.293*	−0.271*	−0.279*	−0.188*	−0.301*	0.428*	0.442*	0.453*	0.460*	0.526*	0.477*	0.924*	0.941*	0.948*	0.949*	0.958*	1

* *p* < 0.05.

Work stressors were moderately and negatively correlated with both humanistic practice ability (*r* = −0.404, *p* < 0.01) and professional benefit (*r* = −0.301, *p* < 0.01). Professional benefit was moderately and positively correlated with humanistic practice ability (*r* = 0.477, *p* < 0.01). Overall, these findings indicate significant linear associations among work stressors, professional benefit, and humanistic practice ability. In addition, professional benefit showed a small indirect effect in the association between work stressors and humanistic practice ability (standardised indirect effect = −0.131; total standardised effect = −0.412).

Work stressors were moderately negatively correlated with humanistic practice ability and professional benefit (*r* values were −0.404, −0.301, *p* < 0.01); professional benefit was moderately positively correlated with humanistic practice ability (*r* = 0.477, *p* < 0.01). The study results indicated a significant and meaningful linear relationship among these three factors. Professional benefit is mediated between work stressors and human practice ability (total standardised effect was −0.412 and indirect effect was −0.131).

### 3.4 Analysis of the Mediating Effect of Professional Benefits Between Work Stressors and the Human Practice Ability of ICU Nurses

**FIGURE 1:**
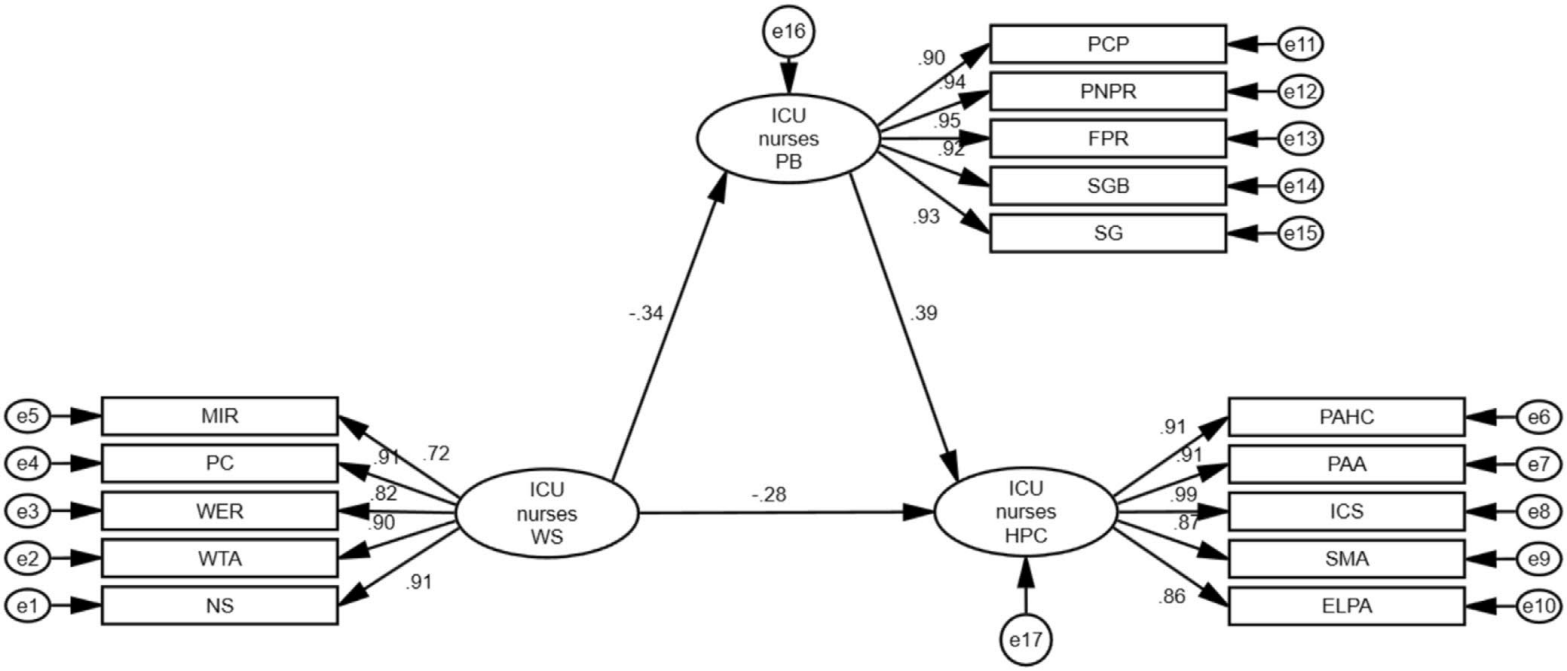
Mediating path of ICU nurse's professional benefits between ICU nurses' work stressors and ICU nurse's humanistic practice ability.

AMOS 24.0 was used for SEM construction and indirect effect testing. Model parameters were estimated using maximum likelihood, and overall model fit was evaluated using CMIN/DF, GFI, AGFI, CFI, IFI, and RMSEA. The initial model showed inadequate fit (CMIN/DF = 3.44, *p* < 0.001; GFI = 0.856; AGFI = 0.801; CFI = 0.954; IFI = 0.954; RMSEA = 0.098). The model was then refined based on MI. Specifically, correlated error terms were specified between SGB and SG, and among PAHC and PAA, SMA, and ELPA. In addition, the covariances e6–e7 and e9–e10 were added. After these post hoc modifications, the revised model demonstrated acceptable fit (CMIN/DF = 1.560, *p* < 0.001; GFI = 0.938; AGFI = 0.912; CFI = 0.990; IFI = 0.990; RMSEA = 0.047), as shown in Figure 1.

Path analysis indicated that professional benefit positively predicted humanistic practice ability (*β* = 0.385, *p* < 0.001), while work stressors negatively predicted professional benefit and humanistic practice ability (*β* = −0.341, *p* < 0.001; *β* = −0.281, *p* < 0.001). The indirect effect of work stressors on humanistic practice ability via professional benefit was examined using bootstrap resampling (5000 samples, 95% CI; Table 4). The standardised indirect effect was −0.131, indicating a small indirect pathway. Given the cross‐sectional design, these findings should be interpreted as associations consistent with the proposed model rather than causal mediation.

**TABLE 4:** Direct, mediating, and total effects of nursing work among 265 ICU nurses.

Mediating path	Effect	Boostrap 95% CI		Effect ratio
		Lower limit	Up limit	
Stressor of nursing work → professional benefits → humanistic practice ability of nurses	Direct effect			
	−0.281	−0.395	−0.162	68.20%
	Mediating effect			
	−0.131	−0.202	−0.071	31.80%
	Total effect			
	−0.412	−0.519	−0.294	

The pathway analysis showed that professional benefit ability had positive prediction (*β* = 0.385, *p* < 0.001); nursing stress nurses had negative prediction (*β* = −0.281, *p* < 0.001); and (*β* = −0.341, *p* < 0.001). The Bootstrap method was used to explore the mediating role of ICU nurses' work stressors and humanistic practice ability, as in Table 4.

## 4 Discussion

### 4.1 Current Status of Humanistic Practice Ability, Nurse's Work Stressors and Professional Benefits of ICU Nurses

In this study, ICU nurses reported a moderate overall level of work stress as measured by the NJSS (70.13 ± 15.70). However, the highest‐scoring domains were management/interpersonal relations and workload/time allocation, indicating that specific stressors were particularly prominent. Consistent with this pattern, humanistic practice ability was at a moderate level overall, whereas lower scores in self‐management and humanistic caring practice may reflect reduced capacity to deliver humanistic care under salient work stressors.

The National Nursing Development Plan of China (2021–2025) emphasises the necessity of strengthening the level of nursing humanistic care. The results of this study indicated that the humanistic practice ability of ICU nurses averaged (105.96 ± 18.26), which is above the average level. This finding aligns largely with previous studies (Yang et al. 2017. ; Zhang et al. 2021, Zhang et al. 2024). This could be attributed to managers focusing more on critical care nurses' emergency treatment capabilities and their acquisition of new technologies like ECMO (Extracorporeal Membrane Oxygenation) and CRRT (Continuous Renal Replacement Therapy), while overlooking the humanistic and social challenges patients confront due to enclosed environments, severe illnesses, and exorbitant medical expenses (Zhang et al. 2021). Analysing the average scores across each dimension, the lowest score was observed in ‘self‐management ability’, followed closely by ‘Practical ability of humanistic care’. This observation is consistent with previous studies (Yang et al. 2017). A possible explanation is that ICU nurses frequently engage in complex emergency care tasks and face heavy learning demands, which leave them with insufficient energy and time to develop their own humanistic practice abilities. In terms of humanistic practice ability, ICU nurses often prioritise the physical care of patients while neglecting their psychological and social needs (Yang et al. 2017). There is insufficient attention given to effective communication with critically ill patients, which can result in unmet psychological needs, such as anxiety and fear. Hospital nursing managers should enhance ICU nurses' understanding and education in humanistic care to continuously improve their awareness in this area. Furthermore, it is essential to strengthen the training of ICU nurses in professional values and career planning to foster a positive professional attitude and systematically enhance their humanistic practice ability.

Most ICU nurses operate in a high‐pressure environment characterised by a substantial workload, which can lead to decreased work motivation, an increased incidence of adverse nursing events, and a higher turnover rate among nursing staff (Li and Liu 2000). The underlying reason could be, partly, that ICU nurses need to engage in effective communication and collaboration with various parties (Ross et al. 2023; Zhang et al. 2024). This study revealed that the total score of work stressors for ICU nurses was 70.13 ± 15.70, indicating a moderate level of stress. Notably, the dimension of ‘Management and Interpersonal relations’ received the highest score at 2.18 ± 0.51 points, closely followed by ‘Workload and Time Allocation’ at 2.10 ± 0.57 points. These findings align with the research conducted by Laurent et al. (Laurent et al. 2021). The sources of this stress may include the necessity for ICU nurses to effectively communicate and collaborate with doctors, colleagues, patients, and their families. During these interactions, differing opinions and expectations can lead to conflicts and misunderstandings, further exacerbating stress levels. Additionally, ICU nurses bear significant responsibilities and must make critical decisions in their daily work, often requiring close observation of critically ill patients. In emergency situations, they must exercise quick judgement and take immediate action. This high‐pressure working environment demands substantial workload and time allocation, significantly increasing the stressors faced by ICU nurses.

The ICU professional benefit score was 61.33 ± 22.21. Among the evaluated aspects, the ‘positive career perception’ score was highest, while the ‘sense of group belonging’ score was lowest. Wang's survey indicated that the ICU career benefit at a middle level is inconsistent; this inconsistency may be attributed to the fact that the study primarily focused on nurses from Shanghai's ICUs, whereas Wang's survey concentrated on ICU nurses in Northwest China (Wang et al. 2023). Differences in patient demographics, the professional‐technical requirements and sampling methods nurses face in these regions may account for the variation in scores. It is noteworthy that ICU nurses' perceived professional benefits are generally low, possibly due to the significant work pressure and challenges they face. In order to improve nurses' perceived professional benefits, nursing management should employ a variety of strategies, such as bolstering professional education, fostering a favourable perception of the nursing profession, and boosting nurses' sense of professional worth. Additionally, a more positive attitude and emotional experience toward their career can be fostered by appropriately raising wage levels to meet nurses' material and spiritual needs.

### 4.2 Correlation Among Humanistic Practice Ability, Work Stressors and Professional Benefit of ICU Nurses

The results of this study indicated a negative correlation between work stress and humanistic practice ability (*r* = −0.404, *p* < 0.01). Specifically, as ICU nurses' stress levels increase, their capacity for humanistic practice declines, aligning with findings from previous studies (Chang et al. 2018; Meixia et al. 2023). This may occur because, under high work pressure, nurses often prioritise addressing clinical challenges over providing psychological and emotional support to patients, thereby diminishing their performance in humanistic practice.

The results indicate a positive correlation between professional benefit and humanistic practice ability (*r* = 0.477, *p* < 0.01). This finding aligns with the research conducted by Zhou  (Zhou et al. 2022) which demonstrates that a heightened sense of professional benefit among nurses corresponds with an improvement in their humanistic practice abilities. Conversely, enhancements in humanistic practice capabilities also contribute to an increased sense of professional benefit. Therefore, it is imperative for medical institutions and management to prioritise the development and enhancement of ICU nurses' professional benefits and humanistic practice skills. By offering essential training and educational opportunities, institutions can strengthen nurses' awareness of humanistic care and communication skills, thereby equipping them to better manage the pressures and challenges of their work. This, in turn, will enhance their sense of professional benefit and humanistic practice abilities, ultimately leading to improved nursing services for patients.

A negative correlation was observed between professional benefits and work stressors (*r* = −0.301, *p* < 0.01). This finding aligns with the results of previous studies (Han et al. 2022; Salminen et al. 2021), likely due to the fact that nurses may experience fatigue and depression in the face of continuous work pressure, making it difficult to derive positive experiences from their roles. This, in turn, impacts their perception of job satisfaction and sense of professional benefit. Specifically, as ICU nurses encounter increased work‐related pressure, their perception of professional benefits tends to diminish. Conversely, when nurses report a higher sense of professional benefits, their work‐related pressure may correspondingly decrease. The intensive workload, high‐stress environment, complex interpersonal relationships, and rapidly evolving medical technology in the ICU contribute to these stressors. Such factors not only adversely affect nurses' physical and mental health but also their overall performance and sense of professional benefit.

Based on the correlation among work stressors, professional benefits, and humanistic abilities, it is suggested that future research could explore strategies to optimise the working environment for ICU nurses. This includes improving career development pathways, enhancing compensation, and other measures aimed at further increasing their professional benefits and humanistic practice abilities, while also mitigating the impact of work‐related stress.

### 4.3 Mediation Effect Analysis of Professional Benefit Between ICU Nurse's Work Stressor and Humanistic Practice Ability

In this study, we constructed structural equation models to examine the mediating effect of professional benefit feelings on the relationship between work stressors and the humanistic practice ability of ICU nurses. The final results indicated that professional benefit feelings served as a significant intermediary in the influence of work stressors on humanistic practice ability, with a total standardised effect of −0.412 and an indirect effect of −0.131. These findings align with previous studies (Han et al. 2022; Salminen et al. 2021) and confirm the critical mediating role of the sense of professional benefit in the context of nurses' humanistic practice ability and work stressors. The total standardised effect of −0.412 suggests that work stressors exert a direct negative impact on humanistic practice ability. Specifically, as nurses experience increased work pressure, their humanistic practice ability may diminish accordingly. This decline may result from excessive work pressure hindering nurses' capacity to maintain high levels of humanistic care and effective communication skills, ultimately leading to suboptimal interactions with patients and families. Furthermore, the indirect effect of −0.131 highlights that professional benefit mediates the relationship between work stressors and humanistic practice ability. In essence, work stressors not only directly impair humanistic practice ability but also indirectly influence it by affecting the sense of professional benefit. As nurses face work pressure, their sense of professional benefit may diminish, consequently impacting their ability to engage in humanistic practices.

Medical institutions and administrators can adopt targeted measures to enhance nurses' sense of professional benefits. First, medical institutions and administrators can provide career development opportunities, such as incorporating humanistic practice competencies into professional title promotions and the selection of outstanding nurses, then the overall humanistic practice capabilities of the nursing team can be improved (Han et al. 2022; Salminen et al. 2021). Secondly, diversified training in nurses' humanistic practice abilities can be strengthened by combining conventional theoretical learning with scenario simulations or online discussions, thereby enriching nurses' humanistic experiences and further deepening their understanding of humanistic care (Mei et al. 2022 ). Finally, the working environment and interpersonal relationships should be improved, such as optimising shift schedules, providing regular psychological support interventions (e.g., mindfulness, resilience, etc.), and fostering strong leadership support and collaborative relationships among colleagues, which can not only alleviate nurses' work pressure but also enhance their humanistic practice abilities, enabling them to deliver higher‐quality nursing services to patients.

### 4.4 Limitations and Recommendations for Future Research

Due to the convenience sampling method employed in this study, which only included ICU nurses from Shanghai, the generalizability of the findings may be limited by regional differences (e.g., hospital resource allocation, cultural background, management models, etc.), resulting in regional and cultural homogeneity. Therefore, caution is warranted when extrapolating these results to populations in non‐metropolitan areas, primary care settings, or different cultural contexts. Future research requiring more precise comparisons should adopt a multicenter prospective cohort design and ensure comparability of samples and measurement instruments. This approach demonstrates our objective and pragmatic scientific attitude. This study used three scales comprising 96 items, which may lead to respondent fatigue, increase human response bias, and affect the authenticity of the data and the reliability of the research conclusions.

Additionally, this study did not conduct stratified or controlled analyses on variables such as years of work experience, shift schedules, and hospital tier, which may influence perceived professional benefit and humanistic practice ability. Future research could further validate these findings through multilevel regression or multi‐group structural equation modelling.

## 5 Conclusion

This study examined the current state of professional benefits, nurses' work stressors, and their humanistic practice ability among ICU nurses. It used a structural equation model to explore how nurse work stressors mediate the relationship between humanistic practice ability and professional benefits. The scores for ICU nurses' professional benefits, work stressors, and humanistic practice ability were 61.33 ± 22.21, 70.13 ± 15.70, and 105.96 ± 18.26, respectively. Nurse work stressors were negatively correlated with humanistic practice ability and professional benefits (*r* values were −0.404 and −0.301, *p* < 0.01); professional benefits were positively correlated with humanistic practice ability (*r* = 0.477, *p* < 0.01). Professional benefits were a mediating variable between nurse work stressors and humanistic practice ability, with a total standardised effect of −0.412 and an indirect effect of −0.131. Medical institutions and managers can implement targeted strategies to improve nurses' perception of professional benefits.

## Funding

This study was funded by: (1) Shanghai Jiao Tong University School of Medicine (SJTUHLXK2023); (2) Shanghai University of Medicine & Health Sciences (SSF‐24‐14‐03); and (3) Shanghai Sixth People's Hospital Affiliated to Shanghai Jiao Tong University School of Medicine (YJHLKT2024‐40).

## Ethics Statement

This study has been approved by the Ethics Committee of the Sixth People's Hospital Affiliated to Shanghai Jiao Tong University School of Medicine (No. 2023‐YS‐135). All methods used in this study were in accordance with the declaration of Helsinki.

## Conflicts of Interest

The authors declare no conflicts of interest.

## Data Availability

The raw data supporting the conclusions of this article will be made available by the authors, without undue reservation.
